# Ultrasensitive Quantification of Crustacean Tropomyosin by Immuno-PCR

**DOI:** 10.3390/ijms242015410

**Published:** 2023-10-21

**Authors:** Mirjana Radomirović, Nikola Gligorijević, Dragana Stanić-Vučinić, Andreja Rajković, Tanja Ćirković Veličković

**Affiliations:** 1 Center of Excellence for Molecular Food Sciences and Department of Biochemistry, University of Belgrade—Faculty of Chemistry, 11000 Belgrade, Serbia; radomirovicmirjana@chem.bg.ac.rs (M.R.); dstanic@chem.bg.ac.rs (D.S.-V.); 2 Center for Chemistry, University of Belgrade—Institute of Chemistry, Technology and Metallurgy, National Institute of the Republic of Serbia, 11000 Belgrade, Serbia; nikola.gligorijevic@ihtm.bg.ac.rs; 3 Ghent University Global Campus, Ghent University, Yeonsu-gu, Incheon 406-840, Republic of Korea; 4 Department of Food Technology, Safety and Health, Faculty of Bioscience Engineering, Ghent University, 9000 Ghent, Belgium; 5 Faculty of Agriculture, University of Belgrade, 11000 Belgrade, Serbia; 6 Serbian Academy of Sciences and Arts, 11000 Belgrade, Serbia

**Keywords:** tropomyosin, immuno-PCR, crustacean allergen, ELISA, shellfish allergen, allergen quantification

## Abstract

Tropomyosin is the major and predominant allergen among shellfish. This study developed an ultrasensitive immuno-PCR method for the quantification of crustacean tropomyosin in foods. The method couples sandwich ELISA with the real-time PCR (rtPCR) amplification of marker DNAs. Monoclonal anti-TPM antibody was the capture antibody, polyclonal rabbit anti-shrimp tropomyosin antibody was the detection antibody, while natural shrimp tropomyosin served as the standard. A double-stranded amino-DNA was covalently conjugated to a secondary anti-rabbit antibody and subsequently amplified and quantified via rtPCR. The quantification sensitivity of immuno-PCR was 20-fold higher than analogous ELISA, with LOQ 19.8 pg/mL. The developed immuno-PCR method is highly specific for the detection of crustacean tropomyosin and is highly precise in a broad concentration range. Tropomyosin recovery in the spiked vegetable soup was 87.7–115.6%. Crustacean tropomyosin was also quantified in commercial food products. The reported immuno-PCR assay is the most sensitive method for the quantification of crustacean tropomyosin and is the first immuno-PCR-based assay for the quantification of food allergen and food protein in general. The described method could be easily adapted for the specific and ultrasensitive immuno-PCR-based detection of traces of any food allergen that is currently being quantified with ELISA, which is of critical importance for people with food allergies.

## 1. Introduction

Food allergies represent a significant health problem of global importance, affecting an estimated 220 million people worldwide [1]. Shellfish has been recognized as one of the eight most common sources of allergens [2]. A high incidence of allergic reactions to shellfish has been more prevalent in the Southeast Asian region, where shellfish constitute a large proportion of the diet [3]. In contrast to other food allergies, in 90% of patients, seafood allergy persists for life. While several proteins have been linked to a shellfish allergy, tropomyosin (TPM) accounts for most of the diagnosed ingestion-related shellfish allergies, with 72–98% of shrimp-allergic patients’ sera showing positive TPM-specific IgE binding [4]. TPM is present both in muscle and non-muscle cells. Together with the troponin complex, it plays an important role in muscle contraction by interacting with actin and myosin [5]. It has a highly conserved amino acid sequence among different invertebrate organisms and thus seems to be a major allergen responsible for cross-reactivity between crustaceans and mollusks, but also other inhaled invertebrates such as house dust mite and insects. Shellfish TPM is a homodimeric coiled coil protein with a molecular weight of 34–38 kDa, depending on the species [2]. Being a heat-stable and high pressure-stable protein, TPM remains mostly intact during food processing conditions and thus poses a major health risk for consumers even after harsh processing conditions, with an even higher percentage of patients showing IgE reactivity to TPM upon the heating of the black tiger prawn extract [2,6].

Avoiding food products containing even traces of allergenic ingredients is still the most efficient treatment for food allergies [4]. In order to inform and protect consumers with allergies, the declaration of allergenic ingredients in prepackaged food is required by law in 66 countries. On the other hand, non-ingredient allergenic components resulting from cross-contamination during manufacturing or packing are not covered by regulations. For the potential presence of unintended allergens, there is now a widespread adoption of voluntary Precautionary Allergen Labeling (PAL) by food manufacturers, providing information on the possible unintentional presence of substances causing allergies. However, mislabeling and the inconsistent use of PAL leads to discrepancy between labeling and the presence of allergens, causing confusion, stress, and uncertainty amongst consumers. As an alternative approach to PAL, the direct quantification of trace amounts of allergens in the food is suggested [1]. Therefore, highly sensitive and specific methods for the detection and quantification of traces of allergens in processed food products are demanded.

Analytical methods for the detection and quantification of food allergens that are currently in use target either allergen, peptide fragment or gene segment coding for a protein of interest. They include either protein-based techniques, such as enzyme-linked immunosorbent assay (ELISA), biosensors, and mass spectrometric methods, or DNA-based methods, such as polymerase chain reaction (PCR) with real-time PCR, enabling precise quantification. Current methods for the detection and quantification of crustacean TPM, based on different sandwich ELISAs, use either both monoclonal antibodies [7], or both polyclonal anti-tropomyosin antibodies [8,9,10,11], or a combination of monoclonal and polyclonal [11,12,13,14]. For the detection of crustacean TPM, different biosensors were developed based on TPM recognition by antibodies [15,16,17,18], as well as by aptamers [19,20,21,22], or mast cells [23,24]. There are also attempts to develop methods for crustacean TPM quantification using other approaches, such as lateral flow immunoassay (LFIA) [25,26,27], via the indirect fluorimetric determination of glutamic acid [28], LC-MS [29,30,31,32,33], as well as real-time PCR [34].

Considering the continuous efforts to improve the current methods as well as to develop new analytical methods for detecting traces of allergens in food, the aim of our work was to develop an immuno-polymerase chain reaction (immuno-PCR or iPCR) method for the detection of crustacean TPM, an important food allergen. The immuno-PCR method, first described by Sano et al. [35], pairs conventional sandwich ELISA with the real-time PCR amplification of the DNA probe linked to the detection antibody and thus combines the antibody specificity of ELISA and the exponential signal amplification of PCR, thereby significantly enhancing the detection limit of conventional ELISA. Furthermore, in comparison to ELISA, a smaller amount of sample is necessary for analysis, and the high sensitivity of iPCR enables the detection of analytes in highly complex samples since the sample material can be significantly diluted, which greatly reduces background effects and increases analysis performance. The immuno-PCR method has so far been used for ultrasensitive detection and the quantification of a wide range of analytes, such as toxins of microbial origin [36,37], viral proteins [38], IgE antibodies [39], etc. However, immuno-PCR has not yet been applied for the detection and quantification of allergens in food samples. Therefore, this study aimed to develop and validate the first immuno-PCR assay for crustacean TPM detection and quantification.

## 2. Results

### 2.1. Development of Sandwich ELISA for Crustacean Tropomyosin Quantification

**Figure 1:**
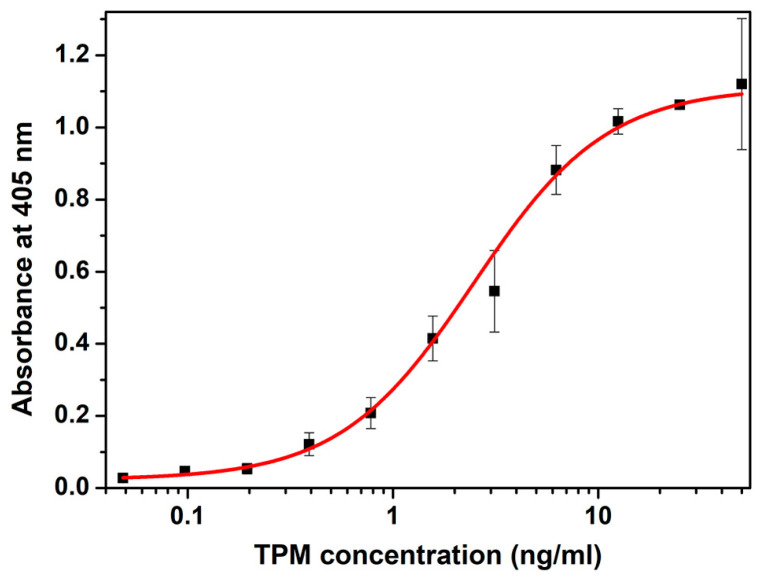
Standard curve of the sandwich ELISA for crustacean tropomyosin quantification.

Sandwich ELISA for TPM quantification was developed using commercially available anti-TPM antibodies. Namely, monoclonal mouse anti-TPM antibody has served as a capture antibody, while polyclonal rabbit anti-shrimp TPM antibody was used as a detection antibody. Alkaline phosphatase-labeled goat anti-rabbit antibody was used as a secondary antibody. Dilutions of each antibody were optimized (not shown). Dilutions presenting the highest signal-to-noise ratio were chosen as optimal. The sigmoidal curve obtained for a serial two-fold dilution of tropomyosin standard from three independent experiments was fitted into the five-parameter logistic model and is shown in Figure 1.

Using the equation of the obtained sigmoidal curve, the LOD and LOQ of ELISA were calculated and were 27.3 pg/mL and 364 pg/mL, respectively. The linear range of developed ELISA was around a 1–6 ng/mL range.

### 2.2. Antibody–DNA Conjugate Preparation

**Figure 2:**
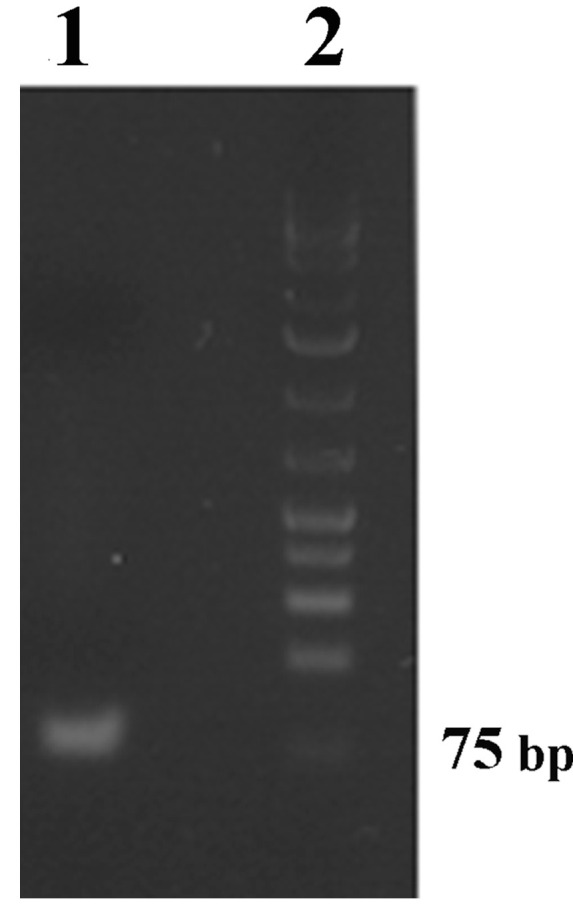
Agarose gel electrophoresis (3% agarose gel) of purified 77-base-pair-long amino-DNA (1) and molecular weight markers (2).

The immuno-PCR format that we propose relies on the use of DNA-labeled secondary antibodies. For an efficient preparation of the antibody–DNA conjugate, large amounts of 77-base-pair-long amino-DNA molecules first had to be synthesized in multiple PCR reactions using the forward primers containing an amino group at 5′ end and synthetic oligonucleotide as template. PCR products were pooled and purified using commercially available PCR clean-up kits. The integrity of the purified amino-DNA molecule was verified by agarose gel electrophoresis, as shown in Figure 2. Purified DNA was concentrated by freeze-drying to obtain the concentration needed for the conjugation reaction. Amino-DNA was conjugated to unlabeled goat anti-rabbit antibody using the commercially available conjugation kit in a 3:1 molar DNA/antibody ratio. Conjugation was performed according to manufacturer’s instructions.

### 2.3. Development of Immuno-PCR for Crustacean Tropomyosin Quantification

**Figure 3:**
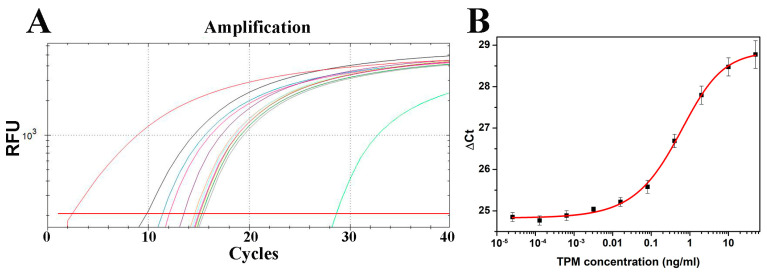
(**A**) Real-time PCR amplification plot obtained for five-fold serial dilution of tropomyosin (TPM). Ten five-fold TPM dilution were used, starting from 50 ng/mL TPM (1) to 2.56 × 10^−5^ ng/mL TPM (10) with curves in black (1), cyan (2), magenta (3), purple (4), orange (5), light blue (6), light pink (7), brown (8), yellow (9), and dark green (10) color. No TPM control is shown in dark gray color. Positive and negative PCR control curves are shown in red and light green color in the far left and far right parts of the plot, respectively. The red horizontal line represents the fluorescence threshold. (**B**) Standard curve of the immuno-PCR for crustacean tropomyosin quantification obtained by plotting ΔCt values against TPM concentration.

We have developed immuno-PCR for TPM quantification analogous to previously developed ELISA. Namely, the same pair of capture and detection anti-TPM antibodies were used as in ELISA. However, instead of using enzyme-labeled secondary goat anti-rabbit antibodies, we used covalent goat anti-rabbit–DNA conjugate previously prepared (Section 2.2). This antibody-bound 77-base-pair-long DNA molecule is further amplified via real-time PCR using the same forward and reverse primers used for its synthesis, only without the amino group at 5′ end of the forward primer. The dilutions of primary antibodies used in immuno-PCR were the same as in ELISA. The dilution of secondary antibody–DNA conjugate was first optimized to obtain the highest signal-to-noise ratio. The obtained real-time PCR amplification plot for the serial five-fold dilution of shrimp TPM standard is shown in Figure 3A. To allow for a direct comparison with ELISA results, obtained Ct values for individual samples were subtracted from the total number of cycles carried out (40) to obtain ΔCt values. Obtained ΔCt values were plotted against TPM concentration. The obtained curve was fitted into a five-parameter logistic model and is shown in Figure 3B.

### 2.4. Method Validation

#### 2.4.1. Sensitivity and Linear Range

Using the equation of the obtained sigmoidal curve (Figure 3B), the LOD and LOQ of immuno-PCR for crustacean TPM quantification were calculated and were 11.3 pg/mL and 19.8 pg/mL, respectively. Compared to LOD and LOQ values obtained for analogous ELISA, immuno-PCR has a 2.4-fold increase in detection sensitivity and 19.8-fold increase in quantification sensitivity. The linear range of developed immuno-PCR was in around the 0.06–2.5 ng/mL range, lower than that of ELISA, with a coefficient of determination of 0.971.

#### 2.4.2. Specificity

**Figure 4:**
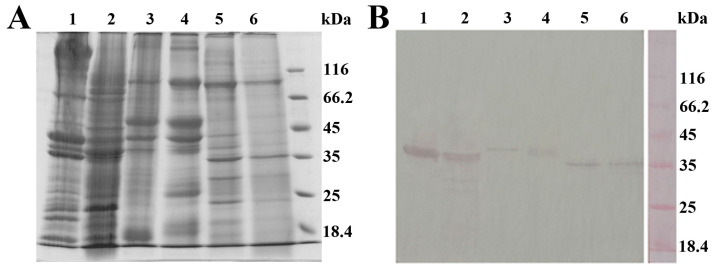
(**A**) SDS-PAGE analysis under reducing conditions (12% polyacrylamide gel, CBB R-250 staining) and (**B**) Western blot analysis of soluble shellfish extracts (1—red shrimp, 2—black tiger shrimp, 3—Mediterranean mussel, 4—blue mussel, 5—clam from Adriatic sea, 6—clam from Yellow sea). The membrane was probed with mouse monoclonal anti-tropomyosin antibodies, followed by incubation with rabbit anti-mouse IgG–alkaline phosphatase conjugate.

Since antibodies determine the assay’s specificity, specificity was tested in the immuno part of the immuno-PCR assay, i.e., analogous ELISA. Specificity was tested on molluscan shellfish, which have about a 55–65% similarity in TPM sequence with crustacean shellfish [4]. The extracts of two crustacean species—red shrimp (*Solenocera melantho*) and black tiger shrimp (*Penaeus monodon*)—and four molluscan species—Mediterranean mussel (*Mytilus galloprovincialis*), blue mussel (*Mytilus edulis*), and two different clams (*Venerupis* spp.)—originating from either the Adriatic sea or Yellow sea were prepared and analyzed in ELISA for tropomyosin content. Protein profiles of extracts were analyzed in parallel via SDS-PAGE, while TPM presence was also analyzed using Western blot under denaturing conditions. In contrast to crustacean shellfish, having 2.5 ± 0.4 mg/g and 3.1 ± 0.1 mg/g of soft tissue of red shrimp and black tiger shrimp, respectively, in all tested molluscan shellfish species, TPM content was under the limit of detection of the ELISA. However, SDS-PAGE protein profiles (Figure 4A) of these two shellfish phyla, followed by Western blot analysis of TPM presence (Figure 4B), show that the amount of TPM in soluble protein extracts is in the same order of magnitude in crustacean and molluscan species. While molluscan TPM is recognized by monoclonal anti-TPM antibodies under denaturing and reducing conditions of Western blot, under native conditions present in ELISA, anti-TPM antibodies specifically recognize only crustacean TPM. This strongly suggests that although tested mollusks have high TPM content, molluscan TPM was not recognized by antibodies used in analogous ELISA, confirming that the immuno part of our immuno-PCR assay is highly specific, efficiently discriminating even TPM from closely related shellfish species.

#### 2.4.3. Precision

The precision of the developed immuno-PCR method was evaluated by inter-day assay variations. As seen in Table 1, RSDs of ΔCt values for inter-assay were from 1.5 to 2.2%, which indicates that the precision of our assay is high and uniform in a broad concentration range covering eight orders of magnitude. These results demonstrate that our immuno-PCR method has high precision, considering that, generally, RSD% between 20% and 30% are regarded as acceptable (10–20% is good, <10% very good).

Additionally, intra-assay variability was also tested using shrimp extract. The extracts of red shrimp (*Solenocera melantho*) were prepared in triplicate and analyzed in three separate immuno-PCR experiments. Calculated RSD% were 8.9, 14.2, and 20.3%, confirming the high method precision of TPM quantification even within real samples.

#### 2.4.4. Method Accuracy

The accuracy of immuno-PCR was validated based on the recovery of TPM after spiking. The accuracy was validated based on recoveries of TPM in vegetable soup as a matrix spiked at three TPM levels—10 LOQ, 50 LOQ, and 100 LOQ, i.e., 0.2 ng, 1 ng, and 2 ng per mL of a matrix, respectively, from which the recovery of TPM was determined in a range of 87.7–115.6% and RSDs were in the range of 5–24.5%. The obtained results are given in Table 2. The results indicated that the method we established has relatively high accuracy.

#### 2.4.5. Food Sample Analysis

To investigate the applicability of the immuno-PCR for TPM quantification, five food products suspected that they might contain crustacean TPM traces, including octopus in sauce, spicy noodles, mild noodles, fish sticks, and tuna pate, were purchased from the local supermarket and used to determine TPM content. Octopus (*Octopus membranaceus*) in tomato sauce and both noodles contained a food allergy disclaimer stating that they might contain crustacean TPM traces, while the other two products are suspected that could contain TPM but did not have such a label. Indeed, our results (Table 2) show that TPM was detected in octopus in tomato sauce and spicy noodles with concentrations of 135 ± 16 ng/g and 116 ± 46 ng/g of the products. TPM was not detected in three other food products, i.e., TPM levels were under the limit of detection.

## 3. Materials and Methods

### 3.1. Materials

Carolina shrimp tropomyosin standard, monoclonal, and polyclonal anti-shrimp tropomyosin antibodies were purchased from Indoor Biotechnologies (product codes: NA-STM-1, MA-1A6, and PA-SHM, respectively; Cardiff, UK). Unconjugated and alkaline phosphatase-conjugated goat anti-rabbit antibodies, alkaline phosphatase-conjugated rabbit anti-mouse antibodies, and low-molecular-weight salmon sperm DNA were purchased from Sigma-Aldrich (St. Louis, MO, USA). Bovine serum albumin was purchased from PAN-Biotech (Aidenbach, Germany). p-Nitrophenylphosphate disodium salt was purchased from Serva (Heidelberg, Germany). Synthetic oligonucleotide and forward and reverse primers were purchased from Integrated DNA Technologies (Coralville, IA, USA). PCR reaction cleanup kit and nuclease-free water were purchased from Qiagen (Hilden, Germany). Oligonucleotide antibody conjugation kit was purchased from Abcam (ab218260, Cambridge, UK). Polycarbonate PCR strips were purchased from Roboscreen (Leipzig, Germany). High protein-binding 96-well plates (NUNC Maxisorp), Taq DNA polymerase, and SYBR Green PCR master mix were purchased from Thermo Fisher Scientific (Waltham, MA, USA). All other chemicals were of analytical grade and purchased from Sigma-Aldrich (St. Louis, MO, USA).

### 3.2. Sandwich ELISA

A 96-well microplate was coated with 100 μL/well capture monoclonal anti-shrimp TPM antibody 1000× diluted in coating buffer (50 mM carbonate–bicarbonate buffer; pH 9.6) and incubated overnight at 4 °C. The remaining binding sites were blocked at room temperature with 300 µL of 1% BSA in Tris-buffered saline containing Tween-20 (TBST; 20 mM Tris buffer, 0.9% NaCl, 0.2% Tween-20; pH 7.4) for 1 h, followed by incubation with tropomyosin standards. A serial two-fold dilution of TPM (initial concentration: 50 ng/mL) was added to the plate for 1 h of incubation. The plate was then incubated with polyclonal rabbit anti-TPM antibody (1000× diluted in 1% BSA in TBST), followed by goat anti-rabbit alkaline phosphatase-labeled antibodies (15,000× diluted in TBST containing 1% BSA). All incubations lasted 1 h at room temperature, each with 100 µL of antibody or tropomyosin standard solution. The wells were washed five times with 300 µL of TBST in between each step. ELISA was visualized with p-nitrophenylphosphate as a substrate (1 mg/mL in 100 mM diethanolamine, 0.5 mM MgCl_2_, pH 9.8). The reaction was stopped with 3 M NaOH after the absorbance at 405 nm reached the value of around 1. The results were fitted to a five-parameter logistic curve. Tropomyosin concentrations were calculated relative to a standard curve.

### 3.3. DNA Preparation and Covalent Conjugation to Secondary Antibodies

A 77-base-pair-long 5′-amino-DNA to be conjugated to secondary antibody was 5′-end aminated via PCR using synthetic oligonucleotide as a template and forward primers containing an amino group at its 5′ end. The sequence of the synthetic oligonucleotide serving as a template was: 5′-TCCGGTCGCTATCGTTTGAAAGTCGAGGGCGACCACGAGGAGGAGGTCTGCGAGGTAGCGTTAATCGAGAGCAGTGA-3′. The sequences of forward and reverse primers were 5′-TCCGGTCGCTATCGTTTGAA-3′ and 5′-TCACTGCTCTCGATTAACGCT-3′, respectively. The PCR mixture contained 1× Taq buffer with (NH_4_)_2_SO_4_, 3 mM MgCl_2_, 0.3 mM each primer, 0.2 mM each dNTP, 2.5 U Taq DNA polymerase, and nuclease-free H_2_O in a total volume of 50 µL. The amplification reaction conditions included denaturation at 95 °C for 4 min, followed by 40 cycles of 95 °C for 20 s, 56 °C for 30 s, and 72 °C for 30 s. The final extension was performed at 72 °C for 5 min. PCR amplification was performed using Mastercycler PCR instrument (Eppendorf; Hamburg, Germany). PCR-amplified amino-DNA was pooled and purified using a PCR clean-up kit. Following purification, amino-DNA was first freeze-dried and then covalently conjugated to secondary unconjugated goat anti-rabbit antibodies using a commercially available oligonucleotide conjugation kit by following the manufacturer’s instructions. The molar ratio of DNA to antibodies was 3:1.

### 3.4. Sandwich Immuno-PCR

Sandwich immuno-PCR was performed using the same pair of capture and detection anti-tropomyosin antibodies used in sandwich ELISA. The assay was carried out in 8-strip PCR tubes that were coated overnight at 4 °C with 50 µL/well of the capture mAb 1000× diluted in coating buffer. Wells were blocked at room temperature with 200 µL of 1% BSA, 0.01% salmon sperm DNA in TBST for 1 h, followed by incubation with standards or samples. The standard curve was prepared by using a five-fold dilution of the shrimp tropomyosin standard, starting from the initial concentration of 50 ng/mL. Strips were then incubated with rabbit anti-shrimp TPM polyclonal antibody and then with goat anti-rabbit antibodies preconjugated with double-stranded amino DNA. All incubations lasted 1 h at room temperature, each with 50 µL of antibody, or tropomyosin standard solution or samples. Strips were washed five times with 200 µL of TBST between each step. Upon incubation with the secondary antibody–DNA conjugate, strips were washed five times with TBST and ten times with nuclease-free water. Secondary antibody-bound DNA was amplified in real-time PCR. Amplification was carried out in a CFX96 real-time PCR system (Bio-Rad; Hercules, CA, USA). The PCR program was as follows: 4 min at 95 °C, followed by 40 PCR cycles of denaturation at 95 °C for 20 s, and 30 s at 56 °C and 72 °C each for annealing and extension phases, respectively. The PCR reaction consisted of 1× SYBR Green PCR master mix and 300 nM primers in a total volume of 50 µL. The same pair of primers was used, except for the forward primer that contained no amino group at its 5′ end. Positive and negative PCR controls were also included. Negative PCR control contained pure master mix only, while positive PCR control had antibody–DNA conjugate added in the amount equivalent to that added to samples. The threshold level was determined to be above the background signal. The threshold cycle (Ct) value was set as the cycle at which the amplification curve of the sample intersected the threshold line. Tropomyosin concentrations were calculated relative to a standard curve.

### 3.5. Food Sample Preparation

For the preparation of spiked samples, the dehydrated vegetable soup was first rehydrated by adding 15 volumes of water to 1 g of dry material. Rehydrated vegetable soup was then spiked with shrimp TPM to obtain the final TPM concentrations of 0.2 ng/mL, 1 ng/mL, and 2 ng/mL. For shellfish extracts preparation and food sample analysis, the extracts of red shrimp (*Solenocera melantho*), black tiger shrimp (*Penaeus monodon*), Mediterranean mussel (*Mytilus galloprovincialis*), blue mussel (*Mytilus edulis*), and two Manila clams (*Venerupis* spp.), originating from Adriatic sea and Yellow sea, and five commercially available food products (octopus in sauce, fish sticks, tuna pate, mild noodles, and spicy noodles) were prepared. Extraction method by Lin et al. [14] was used with minor modifications. Five grams of minced red shrimp or food sample was mixed with 25 mL of phosphate-buffered saline (PBS) containing 1 M NaCl (PBSN), pH 7.4. The mixture was homogenized by three grinding steps of 10 s each using a hand blender. The homogenized samples were centrifuged at 13,000× *g* for 5 min and the supernatant was diluted before testing in ELISA or immuno-PCR.

### 3.6. SDS-PAGE and Western Blot Analysis of Shellfish Extracts

To gain insight into the protein profiles of shellfish extracts, the protein components of soluble extracts were analyzed using sodium dodecyl sulfate polyacrylamide gel electrophoresis (SDS-PAGE) under reducing conditions. Protein components were resolved on 12% polyacrylamide gel and stained with Coomassie brilliant blue (CBB) R-250. TPM presence was confirmed using Western blot. Following SDS-PAGE, proteins were transferred to a nitrocellulose membrane using an electroblotting system (VWR; Darmstadt, Germany). Upon the blocking of non-specific binding with 1% BSA in TBST, the membrane was probed with monoclonal mouse anti-TPM antibody (diluted 3000 times in 1% BSA in TBST). TPM was detected using rabbit anti-mouse IgG conjugated with alkaline phosphatase (diluted 1000 times in 1% BSA in TBST). Immunoblot was visualized with a substrate solution containing 1.5 mg 5-bromo-4-chloro-3-indolyl phosphate and 3 mg nitroblue tetrazolium in 10 mL 100 mM carbonate–bicarbonate buffer containing 5 mM MgCl_2_, pH 9.5.

### 3.7. Limit of Detection and Limit of Quantification Determination

For calculating the limit of detection (LOD) and limit of quantification (LOQ) of ELISA, three or ten times the standard deviation of a series of blanks, respectively, was interpolated on the standard curve in which the absorbance value of the blank was previously subtracted from sample values. For the calculation of immuno-PCR LOD and LOQ, three or ten times the standard deviation of a series of blanks were added to mean of the blanks, and this sum was interpolated on the standard curve.

### 3.8. Precision Calculation

The inter-assay variations were calculated from three replicates of ten concentrations of TPM standard in a range from 2.56 × 10^−5^ to 50 ng/mL, where the same assay was conducted in three days. To evaluate the reproducibility of the developed immuno-PCR method within one day, relative standard deviations (RSDs) of ΔCt values obtained for three independent replicates of the same standard concentrations were calculated in each of the three days (RSD 1 to RSD 3). To evaluate inter-day variations, RSDs of ΔCt values obtained for all nine replicates of the same standard concentration were calculated.

## 4. Discussion

Tropomyosin is a major shellfish allergen responsible for most ingestion-related shellfish allergies [2]. For allergic persons, it is critical to quantify tropomyosin in food products in a specific and reliable manner; therefore, many studies have focused on developing different methods for crustacean TPM detection and quantification, varying in specificity and sensitivity (Table 3). DNA-based methods rely on indirect allergen detection and the absence of DNA does not imply the absence of the protein itself. LC-MS-based approaches are promising, mainly due to an increase in mass spectrometer performances, but require complex sample pre-treatment steps, expensive equipment, and highly trained personnel. Biosensor-based approaches require sophisticated sample preparation and specific/expensive equipment or are otherwise not sensitive enough. TPM analysis based on glutamic acid quantification [28] is not specific enough. On the other hand, due to their high sensitivity and specificity, simplicity, low cost, and high-throughput, ELISA assays are still the most commonly used method for the detection and quantification of food allergens, including crustacean TPM.

In this study, we describe a novel immuno-PCR method for the ultrasensitive and specific quantification of crustacean tropomyosin. To the best of our knowledge, this is the first reported application of immuno-PCR for the quantification of crustacean allergen, food allergen, and allergen in general. Our immuno-PCR assay builds upon the conventional sandwich ELISA format for TPM quantification by coupling it with a real-time PCR amplification of a DNA marker covalently linked to a secondary antibody, thus lowering the detection limit below the commonly used immunological method ELISA. Namely, monoclonal mouse anti-TPM antibody has served as a capture antibody, while polyclonal rabbit anti-TPM antibody coupled to alkaline phosphatase-labeled goat anti-rabbit secondary antibody has served as a detection antibody in our ELISA assay for crustacean TPM quantification, enabling the sensitive detection of shrimp TPM up to 27.3 pg/mL and quantification up to 364 pg/mL. Obtained LOD and LOQ values are comparable to the most sensitive assays for the detection and quantification of crustacean TPM [14,15,16], and up to several orders of magnitude more sensitive than all other assays reported in the literature (Table 3). Interestingly, in our study, as a capture antibody, we used the same 1A6 mAb as was used in these three most sensitive studies, and the same detection pAb used by Lin et al. [14] and Angulo-Ibanez et al. [15].

In our analogous immuno-PCR, an alkaline phosphatase-labeled goat anti-rabbit secondary antibody is replaced with the covalent conjugate of unlabeled goat anti-rabbit antibody and a 77-base-pair-long DNA marker that is amplified in a real-time PCR reaction. Indeed, the subsequent exponential amplification of DNA molecule in real-time PCR has enabled almost a 20-fold increase in quantification sensitivity compared to analogous enzyme-amplified sandwich ELISA, even though half the volume of each assay component was used compared to ELISA (50 µL in immuno-PCR versus 100 µL in ELISA). For this reason, our immuno-PCR method allows for the detection of TPM concentrations that are up to 11.3 pg/mL low, and for the precise quantification of TPM concentrations that are up to 19.8 pg/mL low. Therefore, our developed immuno-PCR assay is the most sensitive assay for the detection and quantification of crustacean TPM, in comparison to all other reported approaches, including the most sensitive one (Table 3).

The lower and wider dynamic range of immuno-PCR allows for the precise quantification of TPM in the 0.06–2.5 ng/mL range compared to that of ELISA, which is in the 1–6 ng/mL range. The dynamic range of immuno-PCR is, therefore, not only more sensitive but, at the same time, enables accurate TPM quantification in the broader concentration range. In addition to being currently the most sensitive method for TPM quantification, the developed method also shows high specificity toward crustacean TPM. Molluscan TPM, although belonging to a closely related shellfish group, is not recognized in our immuno-PCR assay. Developed immuno-PCR shows high precision in a broad concentration range covering several orders of magnitude, while, depending on the spike level, the recovery of TPM in rehydrated vegetable soup as a food matrix was in the 87.7–115.6% range and RSDs were in the 5–24.5% range. Moreover, TPM quantified in the commercially available food products indicates that our highly sensitive and specific sandwich immuno-PCR is efficient in identifying and quantifying crustacean TPM in food samples.

Compared to other recently proposed methods for TPM quantification, our immuno-PCR is thus a rather simple and specific method for TPM quantification that does not require expensive equipment but, most importantly, offers remarkable sensitivity. Although LFIA allows for the rapid on-site detection of allergens at low cost with no requirement for trained operators and well-equipped laboratories, its sensitivity is three orders of magnitude lower than our immuno-PCR assay [25,26,27].

Using mAbs as capture antibodies provides a high level of specificity and selectivity as mAbs bind to a single epitope. On the other hand, pAbs as detection antibodies provide high sensitivity as they recognize multiple epitopes. Therefore, in our immuno-PCR assay, we used mAbs as capture antibodies to obtain high specificity and pAb as detection antibodies to maximize assay sensitivity. Currently existing immuno-PCR assays for the detection of antigens of interest are mostly based either on indirect detection by using streptavidin as a non-covalent linker for biotinylated antibody and biotinylated DNA, or on direct detection by the conjugation of DNA to a detection (primary or secondary) antibody. However, the indirect immuno-PCR non-specific binding of streptavidin or biotin results in highly amplified non-specific signal. Therefore, in our immuno-PCR assay, we have used the direct conjugation of DNA to a secondary antibody to minimize or avoid non-specific binding and decrease the number of steps and washings to increase the assay speed. It is also worth noting that the molar ratio of DNA to secondary antibody in our immuno-PCR assay was set to 3:1. Further increase in the molar ratio of DNA to antibody, if not significantly affecting antibody specificity, would probably lead to an even higher sensitivity of the method. Nevertheless, our immuno-PCR for crustacean TPM quantification serves as a proof of concept for ultrasensitive quantification of any food allergen, either in food samples or in allergen preparations used for emerging oral food immunotherapy. Furthermore, it could be easily adapted for the ultrasensitive quantification of any antigen that is currently being quantified with ELISA, including not only any food allergen, but also food adulterants, food vitamins, and food residues.

## 5. Conclusions

Immuno-PCR for crustacean TPM quantification was developed, enabling the ultrasensitive quantification of TPM in the pg/mL range. Our immuno-PCR method was efficient in identifying traces of TPM in commercial processed food. Since cross-contamination as a consequence of the production process could be a possible source for the involuntary intake of shellfish proteins, investigating TPM levels in food samples is of critical importance for allergic persons. Developed immuno-PCR serves as a proof of concept for the quantification of any food allergen, and allergens in general, offering both specificity and sensitivity far above other methods.

## Figures and Tables

**Table 1:** Immuno-PCR assay inter-day variation expressed as % relative standard deviation (RSD) of ΔCt values obtained for each of the ten concentrations of TPM standard.

TPM (ng/mL)	RSD 1 (%)	RSD 2 (%)	RSD 3 (%)	Inter-Day RSD (%)
50	1.2	1.3	1.7	2
10	0.8	0.2	0.5	1.8
2	0.8	3	1.9	1.6
0.4	0.6	3.8	0.8	1.7
0.08	0.6	0.3	0.6	2.2
0.016	0.4	0.2	0	2.1
0.0032	0.2	0.4	0.4	2.2
0.00064	0.5	0.6	0.1	1.5
0.000128	0.4	0.9	0.7	1.8
0.0000256	0.4	0	0.3	1.7

**Table 2:** Recovery of the method at three spike levels—10 LOQ (0.2 ng/mL of matrix), 50 LOQ (1 ng/mL), and 100 LOQ (2 ng/mL) with relative standard deviations (RSD). Quantification of crustacean tropomyosin in five commercially available products using immuno-PCR. TPM concentration is expressed as ng of TPM per g of product.

Recovery of the Method			
Spike Level	Mean ± SD (ng/mL)	Recovery (%)	RSD (%)
10 LOQ—0.2 ng/mL	0.22 ± 0.04	108.7	19.3
50 LOQ—1 ng/mL	1.16 ± 0.24	115.6	24.5
100 LOQ—2 ng/mL	1.75 ± 0.10	87.7	5
**Quantification of crustacean tropomyosin**			
**Sample**	**TPM (ng/g)**	**Declared by manufacturer**	
Octopus in tomato sauce	135 ± 16	yes	
Spicy noodles	116 ± 46	yes	
Mild noodles	ND	yes	
Fish sticks	ND	no	
Tuna pate	ND	no	

**Table 3:** The literature data on limit of detection (LOD) and limit of quantification (LOQ) for different methods for the detection and quantification of crustacean shellfish TPM; c—capture antibody; d—detection antibody; NA—non-available data; SPE—solid-phase extraction.

LOD	LOQ	Method	Reference
0.4 ng/mL	0.6 ng/mL	ELISA, c: mAb, d: pAb	[11]
0.8 ng/mL	1 ng/mL	ELISA, c: pAb, d: pAb	[11]
6.8 ng/mL	13.67 ng/mL	ELISA, c: pAb, d:pAb	[10]
30 pg/mL	64 pg/mL	ELISA, c: mAb, d: pAb	[14]
2 ng/mL	3 ng/mL	ELISA, c: pAB, d: pAb	[9]
0.71 ng/mL	2.25 ng/mL	ELISA, c: mAb, d: pAb	[12]
3.58 µg/g	7.16 µg/g	UPLC-MS/MS after purification of extracts via immunoaffinity column and digestion by trypsin, with isotope-labeled standard	[32]
72 ng/mL	219 ng/mL	LC-MS/MS after trypsin digestion and SPE, with isotope-labeled standard	[30]
18 ng/mL	59 ng/mL	UHPLC-MS/MS after trypsin digestion and SPE, with isotope-labeled standard	[33]
NA, depends on quality of MS and the removal of impurities	1.6 µg/g	HPLC–MS/MS, after trypsin digestion and SPE, with isotope-labeled standard	[31]
1.3 µM of Glu (about 0.85 µg/mL TPM *)	4.4 µM of Glu (about 2.85 µg/mL TPM *)	Fluorimetric determination of Glu after protein hydrolysis, conversion of Glu to pyroglutamic acid and its derivatization	[28]
0.47 ng/mL	1.6 ng/mL	Electrochemical Immunosensing, c: mAb, d: pAb	[18]
1 µg/mL	2.5 µg/mL	SPR biosensor, c: mAb	[17]
21 pg/mL	70 pg/mL	A biosensor based on a chiral assembly of polymer of gold nanoparticle (AuNP) trimers, c: mAb	[16]
30.76 ng/mL	102.53 ng/mL	Sandwich biosensor based on imprinted magnetic particles and aptamer-modified carbon dots	[22]
0.15 µg/mL	0.5 µg/mL	DNA aptamer assay	[20]
230 pg/mL	1 ng/mL	Visible light-driven photoelectrochemical aptasensor	[19]
0.15 µg/mL	0.5 µg/mL	Mast cell-based electrochemical biosensor	[23]
30 ng/mL	100 ng/mL	Fluorescent magnetic bead-based mast cell biosensor with electrochemical detection	[24]
0.75 ng/mL	NA	ELISA, c: pAb, d: pAb	[8]
0.45 ng/mL	NA	ELISA, c: mAb, d: mAb	[7]
90 pg/mL	NA	ELISA, c: mAb, d: pAb	[13]
25 µg/g	NA	LC-MS after trypsin digestion and SPE, label-free quantification	[29]
Visual 50 ng/mL Instrumental 10 ng/mL	NA	Quantum-dot-based sandwich lateral flow immunoassay, c: pAb	[27]
15.6 ng/mL	NA	Superparamagnetic nanoparticle-based lateral flow immunoassay, c: mAb	[25]
Visual 500 ng/mL Instrumental 50 ng/mL	NA	Quantum-dot-based lateral flow immunoassay, c: pAb	[26]
0.5 ng/mL	NA	Immunochromatographic assay strip, c: mAb, d: mAb	[7]
2 nM (75 ng/mL)	NA	Aptameric biosensor	[21]
47 pg/mL	NA	Amperometric immunosensor based on a sandwich immunoassay, c; mAb, d: pAb	[15]
0.32 ng/mL TPM DNA	NA	Ultrafast PCR	[34]

* Taking into account that TPM has 54 Glu in its sequence.

## Data Availability

The data that support the findings of this study are available from the corresponding author, T.Ć.V., upon reasonable request.
